# Cytotoxic Effect of Immunotoxin Containing The Truncated Form of Pseudomonas Exotoxin A and Anti-VEGFR2 on HUVEC and MCF-7 Cell Lines 

**Published:** 2014-05-25

**Authors:** Elahe Safari, Ahmad Zavaran Hosseini, Zuhair Hassan, Khosro Khajeh, Mehdi Shafiee Ardestani, Behzad Baradaran

**Affiliations:** 1 Department of Immunology, Faculty of Medical Sciences, Tarbiat Modares University, Tehran, Iran; 2 Department of Biochemistry, Faculty of Sciences, Tarbiat Modares University, Tehran, Iran; 3 Department of Medicinal Chemistry, Faculty of Pharmacy, Tehran University of Medical Sciences, Tehran, Iran; 4 Immunology Research Center, Faculty of Medicine, Tabriz University of Medical Sciences, Tabriz, Iran

**Keywords:** VEGFR2, Pseudomonas Exotoxin, Immunotoxin

## Abstract

**Objective:**

Immunotoxins (ITs) have been developed for the treatment of cancer, and comprise of antibodies linked to toxins. Also vascular endothelial growth factor (VEGF) plays a key role in tumor angiogenesis, and the blockade of VEGF receptor-2 (VEGFR2) inhibits angiogenesis and tumor growth. The aim of this study was to produce anti-VEGFR2/rPE (*Pseudomonas exotoxin*) 38 IT to test its cytotoxic activity and mechanism of action.

**Materials and Methods:**

In this basic research and experimental study, at first, DNA that encodes recombinant PE38 protein was inductively expressed in Escherichia coli (*E.coli*) and purified by nickel-sepharose chromatography and further analyzed by western blot. Then, for production of IT, rPE38 was chemically conjugated to anti- VEGFR2. The cytotoxicity response of IT treatment was evaluated by 3-(4,5-Dimethylthiazol-2-Yl)-2,5-Diphenyltetrazolium Bromide (MTT) test in Human Umbilical Vein Endothelial Cell (HUVEC) and Michigan Cancer Foundation-7 (MCF-7) (VEGFR2+) cell lines. The mechanism of IT cytotoxicity was observed by Annexin V staining and flow cytometry. Continuous variables were compared with the analysis of variance (ANOVA; for all groups). P values less than 0.05 were considered statistically significant.

**Results:**

SDS-PAGE showed 98% purity of rPE38 and IT. In vitro dose-dependent cytotoxicity assay demonstrated that anti-VEGFR2/PE38 is toxic to VEGFR2-positive cells. IT treatment significantly inhibited proliferation of HUVEC and MCF-7 in a VEGFR2-specific manner as compared with the control groups (p<0.05). Flow cytometry showed that the mechanism of IT induced cell death is mediated by apoptosis.

**Conclusion:**

IT treatment also caused remarkable synergistic cytotoxicity characterized by decreased cell viability, and an increased apoptotic index by both anti-VEGFR2 and PE38. Thus these results raise the possibility of using anti-VEGFR2/PE38 IT for cancer therapy because nearly all tumors induce local angiogenesis with high VEGFR expression.

## Introduction

Angiogenesis is a complex, highly regulated process that is critical for tumor growth and metastasis (1) and vascular endothelial growth factor A (VEGF-A) is a major regulator for angiogenesis that stimulates vascularization of normal and neoplastic tissues (2). VEGF binds to three receptor tyrosine kinases: VEGF receptor 1 (VEGFR1), VEGFR2 and VEGFR3 (3). VEGF and its receptors are highly expressed in many human cancers.

However, VEGFR2 plays a major role in transducing the angiogenic effect of VEGF on tumor vasculature. Thus it is generally agreed that VEFGR2 on the endothelial cells is the major mediator of angiogenesis in solid tumors and has been an important receptor for a number of anti-angiogenic agents in clinical investigation. Examples of such drugs include chimeric or humanized monoclonal antibodies (mAbs) to VEGFR-2 (4). Overexpression of VEGFR2 was found on activated endothelial cells of newly formed vessels (5). VEGFR2 activation promotes endothelial cell growth, survival and migration, and increases vascular permeability (6). By blocking the signaling of VEGFR-2 with the anti-VEGFR2 antibody, inhibition of tumor vascularization and abrogation of tumor invasion were demonstrated (7). On the other hand, antibody-based therapeutics has been developed to become important constituents for treatment of human malignancies (8).

The efficacy of specific mAbs improve dramatically when conjugated to cytotoxic molecules. These bifunctional chimaeras, known as immunotoxins (ITs), possess considerable potential in cancer therapy since antibody binding to the surface of cancer cells is followed by endocytosis of the antibody-toxin-conjugate. Once internalized, IT treatment induces cell death through two different mechanisms: inhibition of protein synthesis and induction of apoptosis (9). For example, *Pseudomonas exotoxin* (PE) makes an extremely active IT when conjugated to an antibody (10). PE is a 66 kDa single-chain protein containing three disulfide bonds (11) and composed of three structural domains. Domain Ia mediates cell binding (12), domain II is responsible for translocation into the cytosol (13), and domain III catalyzes the adenosine diphosphate (ADP) ribosylation of elongation factor 2 which arrests protein synthesis in eukaryotic cells, causing cell death (14). The function of domain Ib remains undefined, and amino acids 365-380 can be deleted without the loss of cytotoxic activity (15).

Today ITs represent a promising group of targeted therapeutics for cancer patients and many of them are under investigation in clinical trials. Several ITs have so far been made by using mutant forms of PE lacking the native binding domain, which are chemically conjugated to mAbs directed at various "tumor-specific" or normal cellular antigens (16).

In this study, an anti-VEGFR-2/rPE38 IT was produced. At first, the recombinant 38 kDa section of PE (PE38) was produced and then rPE38 was chemically conjugated to the anti-VEGFR2. The antitumor activity and apoptotic effect of the anti-VEGFR2/rPE38 immunoconjugate was investigated in HUVEC and Michigan Cancer Foundation-7 (MCF-7) cell lines. Human umbilical endothelial cell (HUVEC) was selected for the cytotoxicity assay due to high number of VEGFR-2 receptors/cells and MCF- 7 cells were chosen as a breast cancer cell line that expresses VEGFR-2 receptors.

## Materials and Methods

### Cloning, expression and purification of PE38

In this basic research and experimental study, the DNA for PE38 was amplified from *Pseudomonas aeruginosa* *Pseudomonas aeruginosa* O1 (PAO1) by polymerase chain reaction (PCR). The fragment was then cloned into the pET-21a (Qiagen, Name of country, name of company) containing an N-terminal 6-His-tag. PE38 was created using standard PCR and cloning techniques (17). The cloned plasmid sequence was verified by sequencing analysis. Finally, pET-21a-PE38 was transformed into competent *E.coli* BL2. The positive clone was induced with 0.5 mM isopropyl 1-thio-β-D-galactopyranoside (IPTG) (Fluka, Buchs, Switzerland) at a desired cell density (OD 600 nm =0.6). The bacteria were lysed and sonicated after 3 hours culturing. The supernatant and the inclusion bodies were then assessed by SDS-PAGE (18). PE38 was purified by nickel-sepharose chromatography according to the manufacturers’ recommendations (Qiagen, Hilden, Germany). The purified protein was analyzed by 12% SDS-PAGE and then examined by western blot with rabbit anti-His polyclonal antibody. Concentration of purified protein was estimated using bradford protein assay protocol (BPA) (19).

### Immunoconjugation

Human VEGF R2/KDR/Flk-1 Antibody Monoclonal Mouse IgG1 was purchased from R&D system (Minneapolis, MN, USA). PE38 (5-10 mg/ml) in PBS buffer was dissolved in 10 ml Dimethylsulfoxide (DMSO) plus 1 g calcium chloride and stirred at room temperature (RT) for 1 hour. In the other reaction mixture, PE38 was gently mixed with a 10-fold molar excess of acetic anhydride and incubated at RT for 30 minutes. It was then dialyzed against PBS (Please define abbreviation). PE38 (1 mM) was incubated with a 10-fold molar excess of 1-ethyl- 3-(3-dimethylaminopropyl carbodiimide (EDC) (10 mM) (Sigma, St. Louis, MO, USA) and 10-fold molar excess of sulfo-NHS (Nhydroxy- succinimide) (10 mM) (Sigma, St. Louis, MO, USA) for 20 minutes at RT with gentle stirring. To the resultant solution 1 ml of anti-VEGFR2 in PBS buffer (1 mg/ml) was added under gentle stirring, and the obtained solution was incubated for 1 hour for antibody conjugation at RT. The procedure was performed based on the literature with minor modifications (20). Subsequently, the solution was dialyzed against PBS to remove unreacted 1-Ethyl-3- (3-Dimethylaminopropyl) carbodiimide (EDC) and sulfo- NHydroxysuccinimide (NHS). The completion of the conjugation reaction was checked by thin layer chromatography (TLC). Also, this proposal was approved by the Ethical Committee of Tarbiat Modares University.

### Size and zeta potential distribution

Antibody (anti-VEGFR2), protein (PE38) and conjugate (0.5 mg/ml) were checked for any changes in the size and zeta potential distribution before and after conjugation reaction by the dynamic light scattering method (DLS technique) (Malvern, Zetasizer Nano ZS, Worcestershire, UK). Each measurement was carried out in triplicate.

### Cell lines

HUVEC, MCF-7 and fibroblast cells were obtained from Pasteur Institute of Iran.

MCF-7 (human breast cancer cell line) was cultured in RPMI 1640 (GIBCO BRL). Human umbilical vein endothelial cell line (HUVEC) was grown in DMEM (GIBCO BRL). Human fibroblast cells were maintained in DMEM F12 (GIBCO BRL). All of the culture media were supplemented with 10% heat inactivated fetal calf serum, Glutamax, 100 units/ml penicillin and 100 μg/ml streptomycin.

All of the cell lines were incubated at 37˚C in a humidified atmosphere with 5% CO_2_ in a standard tissue culture incubator. All reagents and materials were purchased from Sigma-Aldrich (St. Louis, MO) unless otherwise noted. The confluent cells were detached with 0.01 M trypsin ethylenediaminetetraacetic acid (EDTA).

### Cytotoxicity assay

The proliferation inhibiting activity of IT was determined using a 3-(4,5-dimethylthiazol-2-yl)-2,5- diphenyltetrazolium bromide (MTT; Sigma, St. Louis, MO, USA) colorimetric assay. Briefly, HUVEC, MCF-7 and human fibroblast cells were seeded in a 96-well plate (NUNC, Rochester, NY, USA) (3×10^3^ cells/well), grown for 24 hours and then treated for 24-72 hours with culture media containing various concentrations (from 2 to 20 μg/ml) of anti-VEGFR2/PE38 (conjugated mAb), anti-VEGFR2 non-conjugated mAb and rPE38 (non-conjugated protein), and medium with equal volume of PBS as control. Then 10 μl of the MTT solution (5 mg/ml) was added to each well and the plates were incubated for 4 hours at 37˚C. Following the supernatant removal, the MTT-formazan crystals, formed by metabolically active (viable) cells, were dissolved in 100 μl of DMSO (Sigma, St. Louis, MO, USA). Absorbance at λ=570 nm was recorded using a microplate reader (Bio-Tek Instruments Inc., Winooski, VT, USA). The values for total viability of the treated cells were compared with the values generated for the untreated control cells and reported as the percentage of cell viability. The assays were performed in triplicate and repeated at least three times.

### Apoptosis assay

Apoptosis in the target cells was documented by Annexin V-FITC Apoptosis Detection Kit (BD Bioscience, San Diego, CA, USA). HUVEC, MCF-7 and human fibroblast cells were seeded (5×10^5^ cells/well) in a 12-well plate (NUNC, Rochester, NY, USA). Then, 24 hours after seeding, they were exposed for 48 hours with 10μg/ml anti-VEGFR2/PE38, anti- VEGFR2, rPE38 or PBS (control) at 37˚C, 100% humidity and 5% CO_2_. After 48 hours, the cells were removed from the 12-well plate by incubating with trypsin-EDTA, washing twice in PBS and resuspending in 1 ml of Annexin V-binding buffer at 10^6^ cells per ml. Annexin V-coupled FITC and propidium iodide were added (each at 5 μl per 10^5^ cells). The samples were mixed gently, incubated for 15 minute at RT in dark and then subjected to flow cytometry analysis for apoptosis. The cells were then counted using a BD FACS Canto flow cytometer equipped with BD FACS Diva software (BD Bioscience, San Diego, CA, USA).

### Statistical analysis

Statistical analyses were done by microsoft excel and SPSS software. Continuous variables were compared with the analysis of variance (ANOVA; for all groups). P values less than 0.05 were considered statistically significant.

## Results

### Recombinant PE38 production

DNA encoding the PE38 protein was cloned into pET-21a (+) vector between the *NdeI* (5´) and *NotI* (3´) restriction sites. According to SDS-PAGE, the expression of PE38 protein was strongly induced by addition of 0.5 mM IPTG. The target protein was mainly expressed as soluble protein in E. coli. When the soluble fraction was isolated, the rPE38 protein was purified by Ni -Sepharose chromatography.

**Fig 1:**
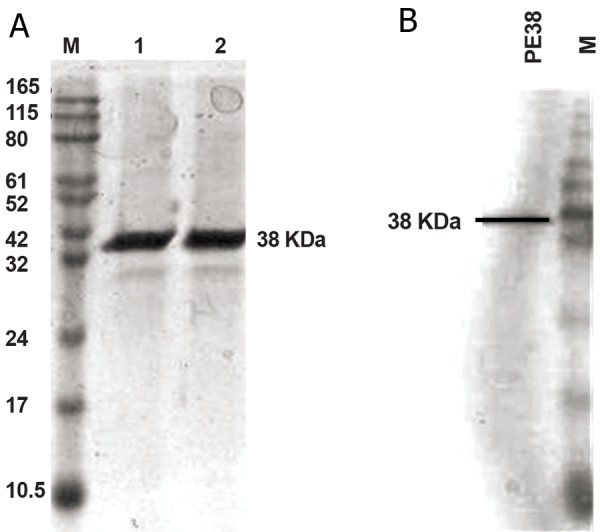
A. Purification of PE38. M. Protein markers, lane 1 and 2. Pooled fraction from Ni-sepharose affinity chromatography. B. Western blot assay. Expression of PE38 was confirmed by western blot with mouse anti-His antibody.

The quantity of the full-length toxins was corrected post-SDS-PAGE analysis (12% [w/v] gel) under reducing conditions using protein standards. The final materials were estimated to be 95% pure, as evaluated by coomassie staining post-SDSPAGE analysis (Fig 1A).

Furthermore, we examined the expression of this protein by Western blot with anti-His antibody and the accuracy of the expressed and purified recombinant proteins was confirmed (Fig 1B).

### Conjugation of anti-VEGFR-2 with PE38

To increase the apoptotic effect of anti-VEGFR2, the rPE38 protein was chemically conjugated to the anti-VEGFR2 and anti-VEGFR2/ PE38 IT was produced. rPE38 was pre-activated to its carboxylic groups by using EDC and NHS, and then reacted with NH2-antibody. The resulting NHS activated PE38 was then covalently linked to anti-VEGFR2. The products obtained from the conjugation reactions were analyzed by TLC. The obtained IT had roughly 98% purity.

### Size and zeta potential distribution

As seen in table 1, when the conjugates were formed from the anti-VEGFR2 and PE38, an increase in the size/polydispersity and a decrease (negative charge) in the zeta potential occurred in the conjugates as contrasted with antibody and PE38.

This may imply that conjugation has happened correctly, the yield of conjugation is good and the conjugate is quite pure.

**Table 1:** Size and zeta potential distributions of the anti VEGFR2, PE38 and their related conjugates


	Size distribution (nm)	Zeta potential (mv)

**Antibody (anti VEGFR2)**	3.4 ± 0.58	-3.35 ± 0.64
**Protein (PE38)**	1.33 ± 1.09	-3.38 ± 0.30
**Conjugate (immunotoxin)**	6.7 ± 0.58	-5.76 ± 0.11


### Inhibition of cell proliferation by the anti-VEGFR2/ PE38 immunotoxin

The concentration-dependent cytotoxic effect of anti-VEGFR2/PE38 was evaluated by the MTTbased colorimetric cell proliferation assay using HUVECs, MCF-7, and human fibroblast cells.

**Fig 2:**
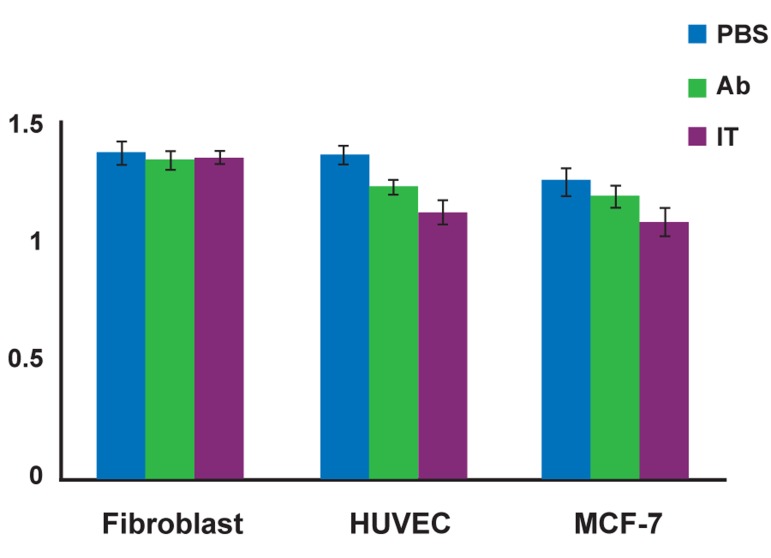
Inhibitory effect of anti-VEGFR-2/PE38 immunotoxin on cells. Cell viability of fibroblast, HUVEC and MCF-7 cells as determined by MTT assays after 48 hours of treatment with PBS (control), anti-VEGFR-2 and anti-VEGFR-2/PE38. The results are presented as mean standard deviation from 3 separate experiments conducted in triplicate for each condition (p<0.05).

After 2 days incubation period with anti-VEGFR2/ PE38, the viability of HUVEC and MCF-7 cells was reduced in a concentration-dependent manner whereas the fibroblast cells remained unaffected (Fig 2).

Anti-VEGFR2/PE38 was active in HUVEC cells [at the concentrations of 10 μg/ml] (Fig 2) while IT had moderate cytotoxic activity toward the MCF-7 [at the concentration of 10 μg/ml]. The fibroblast cells were insensitive to IT. Unconjugated anti-VEGFR2 was less cytotoxic toward HUVEC and MCF-7 cell lines at the concentration of 10 μg/ml. PE38 was not significantly cytotoxic toward any of the cell lines at concentrations of 10 μg/ml (data not shown) (p<0.05). HUVEC cells expressing high level of VEGFR2 were significantly most sensitive to anti-VEGFR2/ PE38 (p<0.05). MCF-7 cells expressing lower level of VEGFR2 than HUVEC cells were significantly sensitive to anti-VEGFR2/PE38 (p<0.05), but the VEGFR2-negative fibroblast cells were insensitive to anti-VEGFR2/PE38. Thus anti-VEGFR2/PE38 significantly decreased cell viability in the VEGFR2 expressing cell lines.

### Induction of apoptosis

We first examined apoptosis as assessed by cell morphology by phase-contrast microscopy. The apoptotic cells displayed cell shrinkage, a rounded morphology and increased detachment.

**Fig 3:**
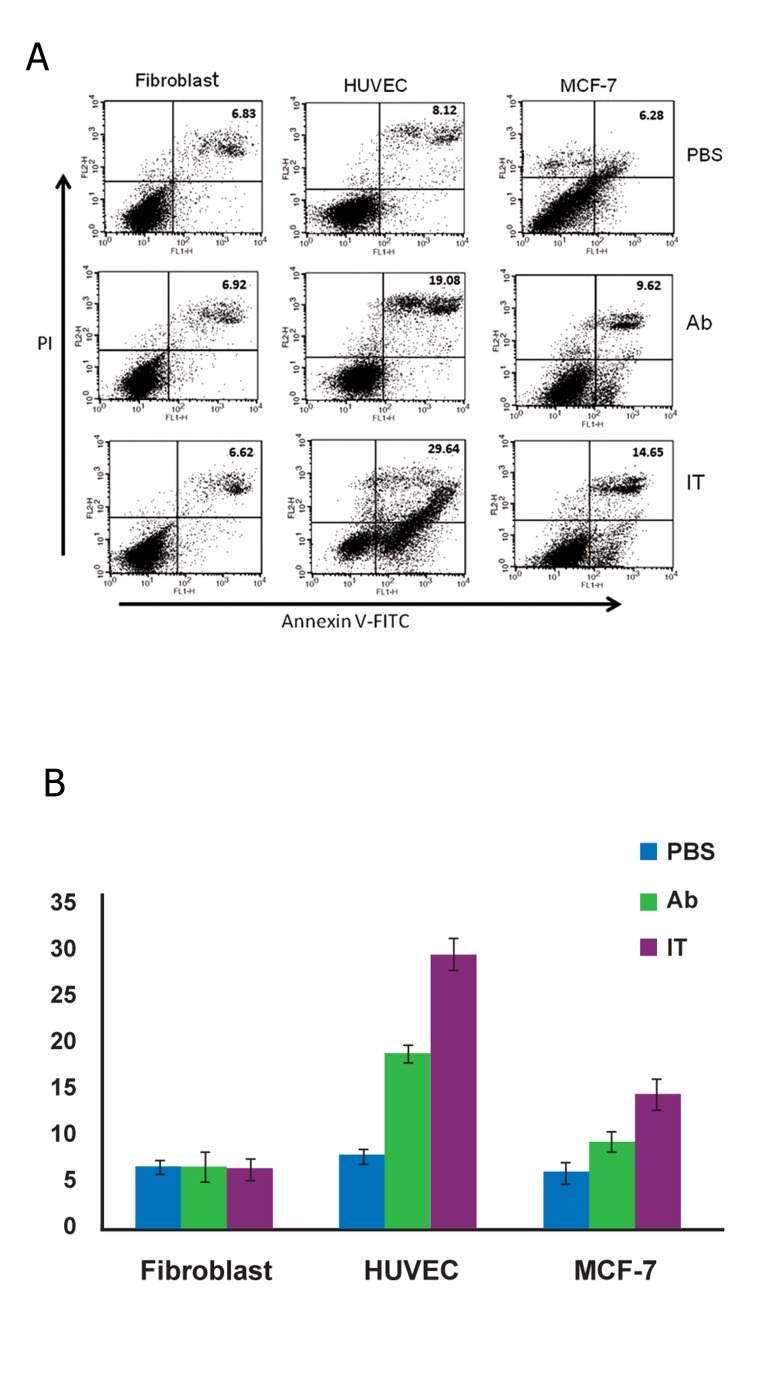
A. Apoptosis as detected by flow cytometry. Cells were incubated with PBS (control) or with of anti- VEGFR-2, anti- VEGFR-2/PE38, (each at 100 ng/mL) for 48 hours. Cells were stained with Annexin V coupled to (FITC) to identify apoptotic cells and propidium iodide (PI) to identify cell nuclei and then subjected to flow cytometry. In each panel, early apoptotic cells are shown in the upper right and late apoptotic cells are shown in the lower right. B. Percentage of apoptotic cells after immunoconjugate treatment. Data are from the flow cytometry experiment in (A). The percentage of cells that were positive for Annexin V staining was calculated. The experiment was repeated three times, with triplicates for each data point. Data are the mean. Error bars=95% CIs (p<0.05).

Apoptosis was detected via Annexin V/PI staining. Anti-VEGFR2/PE38 significantly induced apoptosis in HUVEC and MCF-7 cells as indicated by the reduction of the viable population (Annexin V-/PI) combined with an increase in populations of early apoptotic (Annexin V+/PI) and late apoptotic/dead cells (Annexin V+/PI+) (Fig 3). The fibroblast cells were left unaffected by anti-VEGFR2/PE38.

## Discussion

Inhibition of angiogenesis with antibodies is a central issue in the current strategies for cancer therapy (21,22). Advantages of this approach include applicability to various tumor types (as angiogenesis is required for tumor progression and therapies directed against the tumor vasculature should have broad-spectrum activity), Chance of resistance is low because of the genetic stability of antigen expression on endothelial cells, toxicity effect on normal tissues is low (23). It further reduces the impact of the physical barriers of solid tumors such as heterogeneous blood flow and elevated interstitial pressure, which restricts the penetration and distribution of mAbs through the tumor parenchyma (24). One of the best antigens for vascular targeting is VEGFR2, because it seems to be the most important molecule in mediating the angiogenic stimulation (25). The examples of mAb against VEGFR2 are IMC-1C11 (26) and IMC-1121B (27).

Also it was shown that several tumor types express VEGFRs and inhibition of VEGF (VEGF antisense oligonucleotide) or VEGFRs (neutralizing antibodies) inhibits the proliferation of these cell lines *in vitro*. Thus VEGF is an autocrine growth factor for the tumor cell lines that express VEGFRs (28) and VEGFRs are not specific for endothelial cells and have been localized on several epithelial tumor cells (among them breast cancer) supporting autocrine and paracrine roles for VEGF-A besides angiogenic stimulation (29).

As VEGFR2 is the most important receptor for proliferative activity (30) in VEGFR2-expressing tumors, VEGF inhibition may have dual functions: direct inhibition of tumor cell’s growth and inhibition of angiogenesis (31).

Therapeutic mAbs as well as anti-angiogenic drug are rarely curative by themselves and most of them are administered in combination with chemotherapy (32, 33). Thus there is still an urgent need to enhance the efficacy of antibodies as anti-cancer therapeutics. One solution to this problem is to combine the targeting specificity of mAbs with the tumor-killing potency of cytotoxic effector molecules such as protein toxins to produce immunoconjugates because mAbs kill cells after binding through apoptosis induction (34).

ITs are a better option for those tumors with malignant cells resistant to apoptosis and whose immune systems will not perform antibody or complement-dependent cytotoxicity (35). Radioimmunotherapy is limited by the potency of the radionuclide molecules that can be conjugated to each mAb molecule (36). There are limitations to various surface-targeted strategies but ITs are distinct from these approaches and target the surface of cancer cells with considerable potency, using protein toxins that kill the cells with a single molecule. However, to use toxins as therapeutics, they often have to be modified such as truncated variants of PE (37), which irreversibly inactivates eukaryotic ribosomes.

Various recombinant forms of PE have been made including the one with a MW of 38,000 (PE38) that lacks domain I and has very low liver toxicity. This truncated toxin is nontoxic to the cells but retains the functions of translocation and protein synthesis inhibition when delivered with the targeting molecules (14).

Behdani et al. (38) showed that immunotoxin containing anti-VEGFR2 and PE38 inhibits the proliferation of VEGFR2-expressing cells *in vitro*. However, in this study nonoantibody was used and fused immunotoxin was produced, contrary to our study in which whole antibody was used and conjugated immunotoxin was produced. Similarly, Hu et al. (39) showed that the production of VEGF165-PE38 through gene therapy using a eukaryotic expression plasmid had potential antiangiogenic activity in malignant glioma *in vivo*.

In the current study, an anti-VEGFR2/PE38 was constructed. For this purpose, we first produced recombinant PE38 containing N-terminal 34aa fragment of PE and then coupled the produced rPE38 to the prepared anti-VEGFR2 mAb through chemical conjugation. This conjugation led to a biologically active and cell-type-specific IT. However, production of complex disulfide bonded proteins such as mAb as well as PE38 using recombinant expression systems is challenging and production of properly folded and active bifunctional mAbtoxin fusions is even more difficult. It is, therefore, desirable to produce both proteins separately in active form, followed by covalent coupling to produce the desired conjugate. However, these are first-generation ITs that are relatively primitive molecules, made up of the entire toxin moiety with mutations to render the receptor-binding domain non-functional and are fused to the ligand by chemical means using cross-linking agents to introduce disulfide bonds or establish an amide bond between two proteins. The ligand employed is the whole antibody or monoclonal antibody. The drawbacks of first-generation ITs are: poor tumor uptake; extended half-life and difficulties in production due to their large size.

We produced conjugates of anti-VEGFR2 with the PE38 cytotoxin for studying the *in vitro* inhibition of VEGFR-2 positive cell proliferation. It is known that the mechanism of action of PE38 is based on apoptosis activation.

As mentioned above, series of evidences have elucidated the importance of VEGF signaling not only in vascular cells but also in other cell types and many of tumor cells potentially express VEGFRs. For example, MCF-7 cells were shown to express VEGFR2 (40). Thus HUVEC and MCF-7 cells were selected for the cytotoxicity assay due to their expression of VEGFR2 (41). According to the results of our cytotoxicity assay, anti-VEGFR2/ PE38 could significantly inhibit the proliferation of the cell line overexpressing VEGFR2 in a dose-dependent manner.

As the cytotoxic potency of ITs depends on several properties such as the number of antigens on the cell-surface and the antigen-binding affinity (42), HUVEC cells, which express high number of VEGFR2 receptors/cell were more sensitive to anti-VEGFR2/PE38 than MCF-7 cells, which express moderate number of VEGFR2 receptors/cell. Specificity of the immunoconjugate proteins was also demonstrable by the lack of toxicity to human fibroblasts cells which lack VEGFRs. These data demonstrate that the anti-VEGFR2/PE38 IT is highly toxic only to those cells that overexpress VEGFR-2 receptors.

Also the results of flow cytometry showed that PE38 inhibits cell proliferation by apoptosis. Further, we produced an IT that targets VEGFR2 with therapeutic potential in tumors because angiogenesis is a critical component of tumor growth and metastasis.

## Conclusion

We have developed a new IT and have demonstrated *in vitro* that it has dual inhibitory effect on proliferation of tumor cells and induce apoptosis in them since the developed IT maintained the bioactivities of both anti-VEGFR2 antibody and PE38 toxin. However, the present study had several potential limitations; first, the analyses in this report were done in cultured cell lines and more studies on more cell lines, mouse and at last human are needed. Second, unexpected toxicities may be identified in future researches especially to VEGFR2-expressing normal tissues or cells, and thus preclinical safety evaluation will be needed before clinical development.
